# Acute effects of aerobic exercise on mood and hunger feelings in male obese adolescents: a crossover study

**DOI:** 10.1186/1479-5868-9-38

**Published:** 2012-07-12

**Authors:** Mara Cristina Lofrano-Prado, James O Hill, Humberto José Gomes Silva, Camila Rodrigues Menezes Freitas, Sandra Lopes-de-Souza, Tatiana Acioli Lins, Wagner Luiz do Prado

**Affiliations:** 1 Post Graduate Program of Nutrition, Federal University of Pernambuco, Recife, Brazil; 2 Colorado Center for Health & Wellness, University of Colorado Denver, Denver, USA; 3 Department of Physical Education, University of Pernambuco, Recife, Brazil

**Keywords:** Nutrition, Physical activity, Exercise psychology, Pediatrics

## Abstract

**Background:**

The aim of this study was to determine the acute effects of exercise intensity on anxiety, mood states and hunger in obese adolescents.

**Methods:**

Subjects were eight male obese adolescents (age 15.44 ± 2.06y; BMI 33.06 ± 4.78 kg/m^2^). Each subject underwent three experimental trials: 1) Control, seated for 30 min; 2) Low intensity exercise (LIE) - exercise at 10% below ventilatory threshold (VT); 3) High intensity exercise (HIE) - exercise at 10% above VT. Anxiety (STAI Trait/State), mood (POMS) and hunger (VAS) were assessed before and immediately after the experimental sessions. Comparisons between trials and times were assessed using Kruskal-Wallis and Wilcoxon tests, respectively. Associations between variables were described using a Spearman test.

**Results:**

The largest increase in hunger was observed after LEI (914.22%). Both exercise sessions increased anxiety, fatigue and decreased vigor (*p* < 0.05).

**Conclusions:**

Acute exercise bouts are associated with negative changes in anxiety and mood, and with increases in hunger in obese adolescents.

## Background

Obesity is a global epidemic and is the result of positive energy balance where, over time, energy intake exceeds energy expenditure (EE) [1]. In adolescents, as with children and adults, the treatment of obesity is classically based on lifestyle changes involving increased physical activity and improved diet [2]. Exercise can help in weight loss by facilitating fat loss [3], but, rarely is exercise alone considered to be an effective form of weight control [4]. For exercise to be effective in weight loss, subjects would need to achieve a significant increase in energy expenditure with exercise that was not opposed by increased energy intake. The increase on EE could be followed by increased hunger and energy intake, and this overeating pattern could reverse the negative energy balance provide by exercise [5,6].

Further, exercise is only effective if subjects adhere to the exercise program, and there is some evidence that adherence to exercise is less in obese subjects [7,8]. It is possible that low adherence to exercise might occur in obese subjects via dose-dependent negative impacts of exercise on anxiety and mood [9]. The psychological effects of exercise have been investigated since the early 1970s, and most experts agree that physical activity can enhances the sense of well-being [9].

Classically, the benefits of exercise on mood have been observed in response to moderated aerobic exercise bouts. Moderate intensity exercise typically produces decreases in tension, depression, anger, anxiety and improvements in vigor [10]. On the other hand, there is some evidence that high doses of exercise are associated with negative mood changes that could decrease well-being, and may partially explain the low adherence to the exercise [11].

It is important to note that only few studies have addressed the impact of different types of exercise on mood in obese [12,13], and none have examined this in obese adolescents. Several questions can be asked about the impact of different intensities of exercise on psychological variables in obese adolescents: Are the changes in anxiety, mood and hunger feelings in obese adolescents intensity dependent? Does the acute effect of exercise on anxiety and mood correlate with alterations in hunger feelings in obese adolescents? Answering these questions could help improve our understanding of the optimum behavioral therapies to treat obesity. Thus, the aim of this study was to determine the acute effects of different aerobic exercise intensities on anxiety, mood states and hunger scores in obese adolescents.

## Methods

Volunteers were eight male obese adolescents’ beginners in a long term multidisciplinary obesity intervention program conducted in the outpatient clinic of the University of Pernambuco/Brazil. Adolescents had an average body mass index (BMI) of 33.06 ± 4.78 kg/m^2^, an average age of 15.44 ± 2.06 y, and pubertal stage 3 or 4 using the Tanner scale [14]. The study was approved by the ethical committee of the University of Pernambuco (#154/08). Informed consent was obtained from all volunteers and/or their parents.

Volunteers completed a maximal ergoespirometric test and three experimental (crossover - on seven separate days) sessions:1) control, volunteers remained seated for 2 h; 2) low intensity exercise (LIE), volunteers exercised on a treadmill in an intensity corresponding to 10% below ventilatory threshold (VT); 3) high intensity exercise (HIE), volunteers exercised on a treadmill at 10% above VT. For the LIE and HIE sessions, energy expenditure (EE) was set at 350 kcal, estimated by indirect calorimetry [15]. Each participant served as his own control. Experimental procedures were initiated after three familiarization sessions on treadmill.

During the first visit to the laboratory, anthropometric profiles and body composition were measured, peak oxygen uptake (VO_2peak_) and VT were determined. Obese adolescents were weighed wearing light clothing and no shoes on a Filizola scale (Model 160/300, Brazil) to the nearest 0.1 kg. Height was measured to nearest 0.5 cm by using a wall-mounted stadiometer Filizola (Model 160/300, Brazil). Body composition was determined by bioelectrical impedance (Byodinamics A-310 body composition analyzer) [16].

Oxygen uptake (VO_2_) was measured directly in a continuous incremental protocol on a treadmill (Inbrasport Super ATL, Brazil) as previously described [17]. The inclination was set at 1.0%, the initial speed was 4.0 km/h (four minutes). Thereafter, speed was increased to 1.0 km/h every minute. The termination criteria were the following: volitional fatigue, Borg scale and gas exchange ratio higher than 18 and 1.15, respectively. The greatest VO_2_ obtained before test interruption was considered as VO_2peak_. VO_2_ and carbon dioxide production (VCO_2_) were displayed every 15 seconds using an open circuit respiratory metabolic system (Cortex Biophysik Metalyzer IIB, Germany). Ventilatory threshold was determined by two independent researchers, as the point at which there was a systematic increase in V_E_/VO_2_ without a corresponding increase in V_E_/VCO_2_.

On the other visits, volunteers arrived at the laboratory around 7 AM after an overnight fast, and were given a standard snack 350 kcal (composed of 61.7% carbohydrates, 13.44% proteins and 24.86% lipids), to minimize potential differences in the thermic effect of food (TEF) between subjects. At 7:30 AM, subjects performed the LIE, HIE or control session. After each trial they remained seated for 2 h. The trials were conducted in a controlled temperature room (21–23°C) and in a randomized order, at the same time of the day in order to avoid any circadian variations. Participants were asked to avoid vigorous exercise for 48 h before the sessions.

Spielberger State-Trait Anxiety Inventory (STAI), translated into Portuguese and validated for the Brazilian population [18] was used to measure anxiety. STAI consists of a self rated questionnaire divided in two parts: anxiety-trait (referring to personality traits) and anxiety-state (referring to systemic aspects of the context). Each part has 20 statements. Responses are in a 1–4 scale. Anxiety- state refers to how individuals feel ‘at the moment’, and anxiety-trait to how they ‘generally feel’. Each part varies from 20 to 80 points, and the scores indicate low (0–30), medium (31–49) or high (50 or more) anxiety levels [19].

Profile of Mood States (POMS), translated into Portuguese and validated for the Brazilian population was used to measure mood. Internal consistency for the Brazilian version was reported at 0.62 to 0.91 Cronbach alpha rating [20]. POMS evaluates six mood factors or transitory affective states: Anxiety-Tension, Depression, Anger-Hostility, Fatigue, Vigor, and Mental Confusion. The scores derive from the ratings given to 65 adjectives. Each of the 65 items is scored from 0 (not at all) to 4 (extremely), to detect changes in tension/anxiety, depression, anger, vigor, fatigue and confusion. The final score (Total Mood Disorder) is obtained by subtracting the total of the five factors associated with negative emotions from the only factor of positive emotions (Vigor) [21].

Participants were asked to complete visual analogue scales (VAS) to rate hunger. The questionnaire consisted of six visual analogue scales to rate hunger, fullness, desire to eat, how much you can eat now, urge to eat and preoccupation with thoughts of food. The VAS consisted of horizontal lines of 100 millimeters with defined marks at each extremity that portrayed the expressed desire to eat excessively in the right extremity and have and lack the desire to eat or not hungry in the left extremity [22]. VAS was measured by hand, from left (minimum score of 0 mm) to right (maximum score of 100 mm). In the present study we used only hunger ratings.

Data are presented as median (v.min – v.max). Trials were compared using Kruskal-Wallis test, while differences between basal and acute time points were assessed by Wilcoxon rank test. The relationships between anxiety, mood and hunger feelings were analyzed by Spearman Rank test. Significance was set at *p* ≤ 0.05. For all analysis, statistical power was calculated a posteriori, and in all cases power was greater than 0.80. All analysis was performed in STATISTICA® version 8 for Windows® software.

## Results

As expected, no differences were observed for total energy expenditure between exercise trials, but the LIE session was longer (38.7 ± 7.2 min) with lower treadmill speed (7.2 ± 1.06 Km/h) than HIE (26.1 ± 4.36 min and 8.8 ± 1.30 Km/h, respectively). During low intensity bouts, volunteers exercised at intensity relative to 57.3 ± 12.4% of VO_2peak,_ while that in high intensity was 81.2 ± 11.4% of VO_2peak_. Volunteers’ characteristics are presented in Table 1.

**Table 1:** Characteristics of obese male adolescents submitted to three experimental trials

Variables	Mean (SD)
Age (y)	15.44 (2.06)
Height (m)	1.70 (0.05)
Body Mass (kg)	96.73 (16.99)
BMI (kg/m^2^)	33.06 (4.78)
Fat mass (%)	23.96 (5.15)
Fat mass (Kg)	23.93 (8.53)
VO_2peak_ (ml/kg/min-^1^)	36.4 (6.34)
VO_2_ at VT (ml/kg/min-^1^)	24.1 (6.46)

Note: *BMI* Body Mass Index, *VO*_*2peak*_ peak oxygen uptake, *VO*_*2*_ oxygen uptake, *VT* ventilatory threshold, *SD* Standard Deviation.

**Figure 1:**
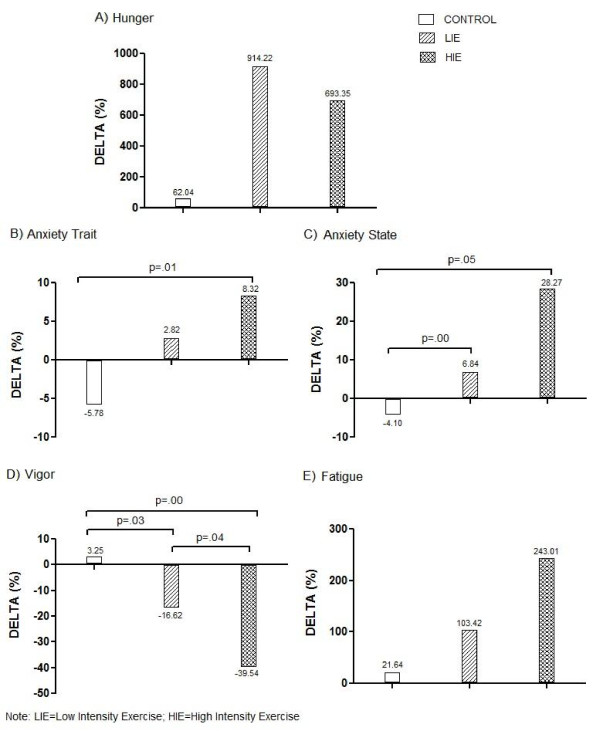
Changes in hunger, anxiety and mood in obese adolescents submitted to three experimental trials.

Hunger score was increased after all trials; however the highest values were obtained in response to LIE. The control session reduced anxiety trait, state and improved vigor. Conversely, high and low exercise intensity increased anxiety trait, state, and fatigue; and decreased vigor. No differences were observed between trials, except for fatigue that was higher after HIE than control session (Table 2). Figure 1 shows the exercise-induced increases in anxiety trait (B), state (C), and fatigue (E), and decreases in vigor (D). Hunger had the biggest changes after LIE, even with a large increase observed after HIE. It is important to note that, no significant associations were seen between hunger, anxiety (trait/state) and mood.

**Table 2:** Hunger, anxiety and mood state of obese male adolescents before and after exercise trials

Variables		Control		Low intensity exercise		High intensity exercise	
		Basal	Acute	Basal	Acute	Basal	Acute
Hunger		1.6(0.4–2.6)	2.1(0.3–5.6)^#^	0.4(0.1–3.9)	2.6(0.1–7.9)^#^	0.4(0.0–4.1)	0.9(0.0–7.7)^#^
STAI	Trait	40.5(25.0–51–0)	37.0(24.0–51.0)^#^	32.5(21.0–66.0)	36.5(24.0–61.0)^#^	32.0(29.0–56.0)	34.0(23.0–55.0)^#^
	State	41.5(20.0–56.0)	40.5(20.0–53.0)^#^	36.5(20.0–66.0)	38.5(21.0–73.0)^#^	31.0(20.0–63.0)	44.0(28.0–64.0)^#^
POMS	Tension and Anxiety	4.5(−4.0–15.0)	1.0(−4.0–17.0)	2.0(−4.0–17.0)	5.5(−4.0–29.0)	−1.0(−4.0–23.0)	9.0(0.0–30.0)
	Depression	3.0(0.0–23.0)	3.0(0.0–30.0)	3.0(0.0–44.0)	2.5(0.0–41.0)	2.0(0.0–32.0)	4.0(0.0–41.0)
	Anger and Hostility	2.0(0.0–26.0)	3.0(0.0–31.0)	3.0(0.0–36.0)	4.0(0.0–42.0)	1.0(0.0–39.0)	4.0(0.0–40.0)
	Vigor	16.0(12.0–25.0)	18.0(10.0–23.0)^#^	15.5(11.0–24.0)	13.5(8.0–22.0)^#^	21.0(7.0–27.0)	12.0(1.0–19.0)^#^
	Fatigue	2.5(0.0–16.0)	4.0(0.0–15.0)	4.5(0.0–18.0)	10.0(5.0–22.0)^#^	5.0(0.0–25.0)	14.0(8.0–27.0)*^#^
	Mental Confusion	0.0(−3.0–12.0)	0.0(−3.0–11.0)	0.5(−3.0–14.0)	−1.0(−3.0–16.0)	−1.0(−3.0–14.0)	0.0(−3.0–15.0)
	Total Mood Disorder	−3.5(−20.0–77.0)	−1.5(−24.0–79)	−4.0(−26.0–106.0)	3.5(−7.0–128.0)	−18.0(−29.0–123.0)	25.0(−10.0–139.0)

Note: *STAI* Spielberger State-Trait Anxiety Inventory, *POMS* Profile of Mood States. Values expressed as Median (v.min- v.max); *vs Control; ^#^ vs Basal; *p* ≤ 0.05.

## Discussion

This study examined the acute effects of aerobic exercise intensity on hunger, anxiety, and mood states in obese adolescents. The main findings of the present study are: 1) High exercise intensity promotes higher negative changes in anxiety and mood than low exercise intensity; 2) Hunger scores are higher after low intensity than high intensity exercise; 3) Even without significant association between anxiety, mood and hunger, both exercise intensities promoted negative changes in these variables.

Research have concluded, based on the hundreds of studies conducted in the last 35 years, that participation in a bout of exercise produces a “feel better” effect [9]. The dual-mode theory suggests that the aerobic-anaerobic transition (anaerobic threshold), may be the point that determines the effect of exercise on mood. Previously, Acevedo et al. [23] reported no relationship between affective valence and physiological variables (heart rate, ventilation, catecholamine release) at or below anaerobic threshold, but affective valence was inversely associated with heart rate and ventilation, in activities that were predominantly anaerobic. For this reason, exercise performed below the VT is pleasant and above the VT is unpleasant.

Acute moderate intensity aerobic exercise has been shown to decrease state anxiety and improve mood state (decreases in tension and depression and increases in vigor) in normal weight individuals [24], it seems that single moderate aerobic exercise bouts “make people feel better” during or after effort. In the present study, exercise performed by obese adolescents either below or above the AT promoted negative changes on anxiety and mood. It seems that unlike normal weight people, obese adolescents do not “feel better” doing exercise, even at low intensities. Ekkekakis and Petruluzzo [25] postulated that believing that all individuals would respond similarly to a given exercise dose (mechanistic model), is too simplistic and ineffective. Based on the underpinnings of hedonic theory, humans generally tend to do what makes them feel better and avoid what makes them feel worse, so that the affective response (how pleasant or unpleasant it is) should influence behavior and decision-making [26]. We suggest that the negative changes reported on mood in obese adolescents can lead to low exercise adherence.

It is possible that the beneficial effects of exercise on well-being are partly or fully mediated by the social interaction when exercise is conducted in a group environment. In that case the effects would have been missed since we conducted the study in a laboratory environment without any social interaction.

Another important finding from this study was that, compared to control session, exercise bouts increased hunger. Further, the greatest increase in hunger was seen after the low intensity exercise. The question of whether exercise, independent of its physiological effects on energy expenditure, influences appetite has long been studied. Several previous studies found that exercise inhibited food intake, hunger and appetite [27-29]. Others found no effects [30,31] or increases [32]. Mackelvie et al. [33] suggested there may be some participants who are compensators and others who are noncompensators, with the latter not increasing their energy intake after exercise. It is possible that these individual differences could explain the contradictories results present in the literature. The factors contributing to differences in energy intake compensation are unknown, but the individual changes in anxiety and mood state in response to physical exercise, could be one of those factors.

Our results show that exercise can increase anxiety (trait/state), decrease vigor and, when performed at high intensity, increase fatigue, in the same time that it enhances hunger. Rutters et al. [34] demonstrated that acute psychological stress was related to an increase in energy intake in the absence of hunger in adults. The authors suggest that differences in energy intake between the stress and control conditions were a function of the differences (delta) in state anxiety scores between immediately after compared to before the stress task, and volunteers who experienced a greater amount of acute stress had the largest increase in energy intake.

A limitation of the present study is use of subjective instruments to assess anxiety, mood and hunger. STAI does not distinguish cognitive symptoms of anxiety (worry, apprehension) from somatic symptoms (tension, nervousness) [9]. In the present study, mood was assessed by a multi-item questionnaire (POMS) that contains a relative large number of items (65 items) [35] and subjects could have become distracted with the time required to complete these items and not paid sufficient attention to the questions, and in some cases, POMS presents a low internal consistency (0.62 Cronbach alpha rating) [20]. It is also important to point out that changes in hunger scores do not necessarily lead to the expected changes in food intake.

The results from this study suggest that while obese adolescents can increase energy expenditure by exercising at high intensity, they tend to show compensatory eating behavior during post-exercise period that will counter the negative energy balance imposed by the exercise. This is followed by negative feelings and a decrease in well-being, that will lead them to avoid regular physical activity in the subsequent periods. In summary, we suggest that obese adolescents do not get pleasure from exercise and that this could be at least partially responsible for low exercise adherence rates often seen in this population.

## Abbreviations

BMI = Body mass index; SD = Standard deviation; EE = Energy expenditure; EI = Energy intake; VO2 = Oxygen consumption; VT = Ventilatory threshold; HIE = High intensity exercise; LIE = Low intensity exercise; VO2peak = Peak oxygen consumption; STAI = Spielberger state-trait anxiety inventory; POMS = Profile of mood states; VAS = Visual analogue scale.

## Competing interests

Authors declare no competing interests.

## Authors’ contributions

MCLF: have been involved in design, data collected, drafting the manuscript and revising it critically for important intellectual content. HJGS, CRMF, SLS and TAL: have been involved in data collected and drafting the manuscript. WLP and JOH: have been involved in design, drafting the manuscript and revising it critically for important intellectual content. All authors read and approved the final manuscript.
